# Thymic Stromal Lymphopoietin and Tezepelumab in Airway Diseases: From Physiological Role to Target Therapy

**DOI:** 10.3390/ijms25115972

**Published:** 2024-05-29

**Authors:** Diego Bagnasco, Laura De Ferrari, Benedetta Bondi, Maria Giulia Candeliere, Marcello Mincarini, Anna Maria Riccio, Fulvio Braido

**Affiliations:** 1 Allergy and Respiratory Diseases, IRCCS Policlinico San Martino, University of Genoa, 16132 Genoa, Italy; laura231283@hotmail.it (L.D.F.); bennina.bondi@gmail.com (B.B.); giuliacandeliere@gmail.com (M.G.C.); marcello.mincarini@hsanmartino.it (M.M.); anna.maria.riccio@unige.it (A.M.R.); fulvio.braido@unige.it (F.B.); 2 Department of Internal Medicine (DIMI), University of Genoa, 16132 Genoa, Italy

**Keywords:** severe asthma, TSLP, epithelium, CRSwNP, mechanisms, alarmins, Tezepelumab, T2 inflammation, cytokines

## Abstract

Thymic stromal lymphopoietin (TSLP), is a protein belonging to a class of epithelial cytokines commonly called alarmins, which also includes IL-25 and IL-33. Functionally, TSLP is a key player in the immune response to environmental insults, initiating a number of downstream inflammatory pathways. TSLP performs its role by binding to a high-affinity heteromeric complex composed of the thymic stromal lymphopoietin receptor (TSLPR) chain and IL-7Rα. In recent years, the important role of proinflammatory cytokines in the etiopathogenesis of various chronic diseases such as asthma, chronic rhinosinusitis with nasal polyposis (CRSwNP), chronic obstructive pulmonary diseases (COPDs), and chronic spontaneous urticaria has been studied. Although alarmins have been found to be mainly implicated in the mechanisms of type 2 inflammation, studies on monoclonal antibodies against TSLP demonstrate partial efficacy even in patients whose inflammation is not definable as T2 and the so-called low T2. Tezepelumab is a human anti-TSLP antibody that prevents TSLP-TSLPR interactions. Several clinical trials are evaluating the safety and efficacy of Tezepelumab in various inflammatory disorders. In this review, we will highlight major recent advances in understanding the functional role of TSLP, its involvement in Th2-related diseases, and its suitability as a target for biological therapies.

## 1. Introduction

In recent years, the important role of proinflammatory cytokines in the etiopathogenesis of several chronic diseases such as asthma, chronic obstructive pulmonary diseases (COPDs) [1,2,3], chronic rhinosinusitis with nasal polyposis (CRSwNP) [4,5,6,7], and atopic dermatitis [8,9] has been investigated. Thymic stromal lymphopoietin (TSLP) is a protein belonging to a class of epithelial cell-derived cytokines commonly called alarmins, which also includes interleukin (IL)-25 and IL-33. It is a pleiotropic cytokine, first identified in tissue culture supernatants of a murine thymic stromal cell line, as a growth factor capable of inducing the proliferation and differentiation of pre-B cells in the absence of IL-7 with which it shares overlapping functions [10]. The human TSLP gene is located on chromosome *5q22.1* next to the atopic cytokine cluster on *5q31*. It has a genomic sequence of approximately 740 bp and encodes a protein of 159 amino acids [11]. Two variants for TSLP in human tissues have been discovered: the main variant expressed in steady state is the shorter isoform (sf TSLP), which plays a homeostatic role; it is an antimicrobial protein displaying antibacterial and antifungal activity against *B. cereus*, *E. coli*, *E. faecalis*, *S. mitis*, *S. epidermidis*, and *C. albicans* [12]. The longer form (lfTSLP) is upregulated in inflammatory conditions. TSLP expression can be induced by a variety of inflammatory cytokines and TLR ligands, predominantly by dendritic cells (DCs), gut and lung epithelial cells, and skin keratinocytes [13].

### TSLP Inflammation-Related Diseases and Tezepelumab

Elevated levels of TSLP seem to be associated with asthma and atopic dermatitis, which are usually both T2 inflammation-mediated conditions. It is believed that the chronic overexpression of TSLP may result in an increased sensitivity to allergen [14]. Moreover, TSLP could also be related to COPD pathogenesis [3]. Initially, specific T2 cytokines such as IL-4, IL-5, and IL-13 have been studied to develop drugs able to inhibit or regulate their biological activity [15]; then, given the consolidated role of alarmins in epithelial-derived inflammation and the search for a possible alternative to the above-mentioned cytokines target, research was on drugs against alarmins, particularly TSLP. The first marketed drug against alarmins is Tezepelumab, an IgG2λ monoclonal antibody developed with the goal of modulating type 2, and to some extent non-type 2, inflammation [16]. This biological treatment can inhibit the action of TSLP by binding to it and preventing in turn its binding to its heterodimeric receptor TSLPR [17]. Tezepelumab has recently been approved for the treatment of patients with severe asthma [18,19] and is being studied for patients with other diseases such as CRSwNP and COPD. Trials on the efficacy of Tezepelumab not only demonstrate an expected efficacy in T2 patients but also interestingly a partial effect in patients whose inflammation is not definable as T2, devoid until now of available biological treatments [20].

## 2. Methods

In this review, we will provide an overview of recent investigations that have analyzed clinical and molecular features, useful in understanding the functional role of TSLP, its involvement in Th2-related diseases, and its suitability as a target for biologic therapies. Moreover, we will highlight recent evidence on Tezepelumab treatment outcomes in asthma, COPD, and CRSwNP with the aim of offering a comprehensive analysis of the existing literature within this field of study, identifying current gaps or problems, being critical and constructive, and providing recommendations for future research. We use original articles, reviews, and meta-analyses from literature, focusing principally on documents from the last 10 years or, if older, considerably crucial for the drafting of the manuscript. The source of the literature was PubMed, using TSLP and mechanisms on respiratory diseases both physiological and pathological, as keywords and referring to both in vivo and in vitro articles. Finally, we again used PubMed and ClinicalTrials.gov to search what was in the literature, or under study, regarding clinical trials performed on Tezepelumab for the diseases that were addressed in our review.

## 3. TSLP in Epithelium-Driven Airways Inflammation

**Figure 1:**
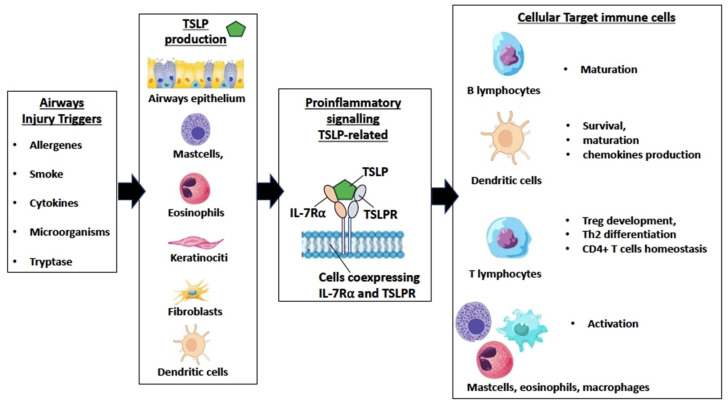
Schematic representation of key points in the production of thymic stromal lymphopoietin (TSLP), proinflammatory signaling by the ternary TSLP receptor-TSLP-Interleukin-7 Receptor alpha (TSLPR-TSLP-IL-7Rα) ternary complex and related effects on target cells.

Regarding TSLP production in the epithelium, a multitude of triggers including allergens; cigarette smoke; cytokines (e.g., TNF-α, IL-1β); viral, bacterial (e.g., *Staphylococcus aureus*), and fungal products; tryptase; and mechanical injury can activate lung and gut epithelial cells and keratinocytes to release TSLP [21,22]. TSLP signaling complexes operate via a cooperative stepwise mechanism on the surface of cellular targets. TSLP plays its role by binding to a high-affinity heteromeric complex, composed of thymic stromal lymphopoietin receptor (TSLPR) chain and IL-7Rα. In particular, TSLP, which is positively charged, binds to the thymic stromal lymphopoietin receptor (TSLPR), which is negatively charged, with high affinity and fast kinetics; subsequently, IL-7Rα associates with the TSLPR-TSLP binary complex to create the ternary TSLPR-TSLP-IL-7Rα complex [23]. This receptor complex, on cells co-expressing TSLPR and IL-7Rα, phosphorylates Janus Kinase (JAK) and signal transducers and activators of transcription (STAT) 5 to initiate proinflammatory signaling. Various immune cells have been reported to express TSLP receptor (TSLPR), including dendritic cells (DCs), basophils, CD4 + T cells, and group 2 innate lymphoid cells (ILC2s), and different cell types have been proposed to have an essential role in TSLP-mediated type 2 inflammation [24]. TSLP exerts a deep influence on the polarization of dendritic cells to drive T helper (Th) 2 cytokine production. TSLP also promotes T cell proliferation directly in response to T cell receptor activation and Th2 cytokine production and supports B cell expansion and differentiation. TSLP further amplifies Th2 cytokine production through mast cells and natural killer (NK) T cells [25] (Figure 1).

Recent studies have focused on the analysis of the gene expression of DCs, activated with different cytokines (TSLP, CD40), to identify the mechanism by which TSLP induces cells to differentiate into TNF-alpha producers and to polarize towards TH2. From the analysis, it has been shown that TSLP induces DCs to express a protein of the TNF superfamily: OX40L [26]. It has been demonstrated that the neutralization, by antibodies, of this protein, leads to the inhibition of interleukins, correlates with the TH2 profile including TNF, and increases the production of IL-10. Thus, by treating naïve T lymphocytes with OX40L, they extend TNFα production and reduce IL-10 production: in this way, OX40L induces a polarization toward type 2 inflammation. Furthermore, the function of this ligand is performed in the absence of interleukin 12. In the presence of this, however, OX40L loses the ability to polarize cells towards the TH2 profile. IL-4 has a synergistic effect with OX40L in the development of the TH2 response [27].

### 3.1. TSLP, Alarmins, and Epithelium

With TSLP being produced by the epithelium, it is interesting to analyze the different types of epithelia in airways. Along the whole respiratory tract, the epithelial lining changes in order to accommodate different functions. The nasal cavity includes various types of epithelia and in the area of the inner nasal valve, the multi-layered squamous epithelium passes into a multi-row cylindrical epithelium [28]. Referring to the lung epithelium, in the large airways, the major cell types are ciliated, undifferentiated columnar, secretory, and basal cells. In the small airways, the cell types are similar, with relatively more ciliated cells, and the secretory cells shift to the Clara cell type. The airway epithelium then merges with the alveolar epithelium, with type I and type II cells. Moreover, a variety of less common cell types, such as cartilage cells and mucus glands in the large airways, and neuroendocrine cells are also present [29]. In daily contact with the environment, the airway epithelium plays the role of the first line of airway defense against pathogens and antigens. It has a close cross-talk with cytokines and inflammatory proteins with a defensive role. The overexpression or dysregulation of epithelial cells is proven to be involved in the etiopathogenesis of inflammatory diseases [30]. The principal cells and cytokines produced by the airway epithelium, or implicated in its effect on inflammation, are ILC2 cells and alarmins (IL-25, IL-33, and TSLP) [30,31]. Alarmins, in turn, induce ILC2 production. ILC2s, along with TH2, are a pivotal part of the innate immune system and can play a crucial role in the induction of type 2 inflammation [32]. The action of ILC2 cells promotes the production of several cytokines, such as IL-4, IL-5, IL-9, and IL-13, which in turn are implicated in the production, maturation, and development of the eosinophils, basophils, mast cells, B cells, immunoglobulin (Ig) E, main cells, and cytokines of T2 inflammation damage [33]. Alarmin production by fibroblasts, endothelial cells, and epithelial cells, is promoted by the action of goblet cells and occurs through contact between the respiratory epithelium and antigens. IL-25 is expressed through lung epithelial and hematopoietic cells [34] and carries out its action by binding to its receptors IL-17RA and IL-17RB, which then elicit its functional responses [35,36]. Therefore, IL-25 acts as a modulator of both the innate and the adaptive immune system through the activation of ILC2 and Th2 cells in a manner that evokes Th2-type mucosal inflammation [37]. IL-33, in the lung, is produced by bronchial epithelial cells, and if upregulated, it stimulates the production of ILC2, which in turn produces IL-13, promoting type 2 inflammation [37,38]. TSLP stimulates the activation of mast cells, immature DCs, basophils, eosinophils, and lymphocytes [39,40] and also plays a role in neutrophilic inflammation by inducing, through the activation of DCs, the polarization of naïve T cells toward a Th17 phenotype, which subsequently releases IL-17, thereby promoting not only T2 but also non-T2 inflammation [41].

### 3.2. TSLP Production and Airway Remodeling

In addition, the release of TSLP leads to a proliferation of the bronchial epithelium and nasal epithelium through the activation of fibroblasts, which leads to smooth muscle hypertrophy and increased remodeling, typical of advanced stages of asthma and nasal polyposis [42]. Similar to what happens in bronchial remodeling and high airways, particularly the nasal epithelium, when stimulated by antigens and allergens, produces cytokines such as IL-25, IL-33, and precisely TSLP, which, together with other cytokines such as IL-1, IL-6, and TNF-α, promote the activation of fibroblasts that proliferate and over-accumulate at the basement membrane level. This deposition and production of the cytokines just mentioned promote the secretion of proinflammatory cytokines and chemotactic factors, driving nasal remodeling and polyps generation [43].

### 3.3. TSLP in Asthma and COPD

In recent years, therefore, TSLP has been the subject of research aimed at deepening and expanding the current knowledge on the physiological and pathological pathways in which it is involved. The cross-talk between the external and internal environment, in the respiratory tract, involves a macrophage/dendritic cell transepithelial network. Canè et al. recently investigated TSLP localization via the Western blot, immunofluorescence, and confocal microscopy of macrophages isolated from human lung parenchyma (HLMs), which were also activated by T2-high (IL-4, IL-5, and IL-13) and T2-low (lipopolysaccharide: LPS) immunological stimuli. The study provides novel findings demonstrating for the first time the constitutive presence of TSLP in HLMs cytoplasm. This novel observation highlights a striking difference between two major alarmins, TSLP and IL-33, and evidence of a novel immunologic circuit between HLMs and TSLP. Given the central role of macrophages in airway inflammation, this autocrine loop holds potential translational relevance in understanding the innovative aspects of the pathobiology of asthma and COPD [3]. Paplinska et al. gave birth to the first study that evaluated the impact of interactions between human monocyte-derived dendritic cells (moDCs) and epithelial cells co-cultured with monocyte-derived macrophages (moMφs) on TSLP, IL-33, and IL-17A expression in asthma and COPD, concluding that TSLP, IL-33, and IL-17A expression in moDCs are differently regulated by the epithelium in asthma, COPD, and healthy subjects. The study used a triple-cell co-culture model, utilizing nasal epithelial cells obtained with nasal brushing, along with moMφs and moDCs. In the asthma group, researchers have found an elevated TSLP mRNA expression in moDCs co-cultivated with both epithelium and moMφs, compared to moDCs alone. However, only the difference between moDCs and moDCs/epithelium + moMφs was significant. The asthmatic moDCs were characterized by the highest TSLP mRNA expression in di- and triple-co-cultures compared to their respective COPD groups. In the COPD groups, there were no differences in TSLP mRNA expression in COPD groups regardless of the co-culture type used [44]. Moermans et al. investigated the sputum IL-25, IL-33, TSLP, IL-23, and IL-36 in healthy controls, asthmatic, and COPD patients. There was no correlation between any sputum cell type and TSLP gene expression in the whole cohort. However, in obstructive airway diseases, TSLP expression appeared negatively correlated with sputum eosinophil absolute value (r = −0.51, *p* < 0.05), and a trend was observed for a correlation with eosinophil percentage (r = −0.39, *p* = 0.07) [45]. A multicenter study analyzed serum levels of IL-25, TSLP, and eosinophils in relation to COPD exacerbation risk in the KOCOSS cohort, a multicenter COPD cohort created by 54 medical centers in South Korea. Patients with higher TSLP levels exhibited poorer exercise capacity. The TSLP-low group was younger (67.8 ± 7.4 vs. 69.4 ± 7.7 years, *p* = 0.02), and individuals in the group were more likely to be current smokers (34.9% vs. 6.3%, *p* = 0.04). There were no differences in the symptom scores; however, the 6MWT exercise capacity was poorer in the TSLP-high group (422.7 ± 106.0 vs. 400.6 ± 106.5 m, *p* = 0.03). The FEV1 did not differ between the two groups; however, the FEV1/FVC and DLCO were lower in the TSLP-high group (53.6 ± 11.4 vs. 51.6 ± 11.6, *p* = 0.04; 67.7 ± 18.0 vs. 63.1 ± 18.4%, *p* = 0.01, respectively). There were no differences in the levels of Th2 inflammatory biomarkers or the numbers of prior exacerbations. A high TSLP level was associated with a lower risk of severe exacerbation but only in the eosinophil-low group [46]. In a prospective cross-sectional study, Nejman-Griz et al. measured serum and induced sputum levels of periostin, TSLP, IL-4, and IL-13 in 12 atopic asthmatic patients, 16 COPD sufferers, and 10 controls. In general, the levels of all the investigated cytokines were higher in the serum than in the induced sputum. The smallest (2-fold) difference between serum and induced sputum concentrations was found for TSLP. The absolute concentrations of periostin and TSLP were higher in patients with asthma compared to patients with COPD and controls [47].

The long and short forms of TSLP seem to differentially regulate IgA production, which may help explain the mechanisms behind the development and exacerbations of asthma and support the effectiveness of therapeutic interventions targeting aberrant TSLP production. Van Heerden et al. investigated how homeostatic short TSLP (shTSLP) and asthma-associated long TSLP (loTSLP) regulate IgA production using irradiated CD40L-expressing L cells and cytokines to stimulate B cells from healthy donors under T cell-dependent conditions. Results showed that loTSLP but not shTSLP inhibits the production of IgA via memory B cells. The effect of loTSLP was selective for IgA and was not observed for IgM, IgE, or IgG1-4. Retinoic acid also promotes the production of IgA in the presence of loTSLP and may, thus, be able to restore IgA production in asthma patients in the presence of aberrant TSLP signaling. The addition of shTSLP or loTSLP did not affect B cell proliferation, neither in the presence nor the absence of CphosphodiestericG (CpG) [48].

Concerning COPD, it is well known that respiratory viruses are triggers of the exacerbation of chronic airway inflammatory diseases and are closely associated with disease exacerbations, hospitalizations, and significant morbidity and mortality. Well-differentiated primary bronchial epithelial cells (WD-PBECs) from severe COPD and age-matched healthy controls and cultured in the air–liquid interface (ALI) model allowed for the evaluation of differentiation phenotype, epithelial barrier integrity, pathological response, and cytokine-secreting profile, before and after HRV infection. With respect to healthy controls, a Th1/Th2 imbalance and a strong interferon and proinflammatory cytokine response were observed in COPD cultures. In COPD cultures, decreased TSLP and IL-13 cytokine levels prior to HRV infection were observed [49].

### 3.4. TSLP in CRSwNP

Finally, regarding CRSwNP, TSLP was certainly elevated in past studies. In contrast, IL-25 and IL-33 were not always elevated in NPs [50]. As well as the epithelium of the lower airways, that of the nasal cavities also expresses TSLP through PAR-2, IL-33, and IL-25 signaling in response to exposure to allergens, viruses, bacteria, and irritants [51,52]. Building on the mechanisms just mentioned, we also see how the action of TSLP stimulates increased ILC2 production, as already shown in bronchial asthma patients, and also in the upper airways. Studies in patients with CRSwNP have shown that increased DNA methylation, at the TSLP locus, is associated with the pathogenesis of CRSwNP and that TSLP expression itself is increased in these patients, thus confirming its role in the pathogenesis of the disease [53,54]. Ogasawara et al. evaluated the role of the receptor activator of NF-κB (RANK) ligand (RANK-L), a member of the TNF superfamily, and found it significantly elevated in nasal polyps (NPs). Data have also shown the ability of an agonistic antibody against RANK to induce the production of type 2 cytokines in human ILC2s, and TSLP significantly enhanced this reaction [55]. Again, as already described, TSLP is related to the expression of OX40L and is increased in patients with CRS compared with those without it [56].

## 4. Tezepelumab in Airway Diseases

**Figure 2:**
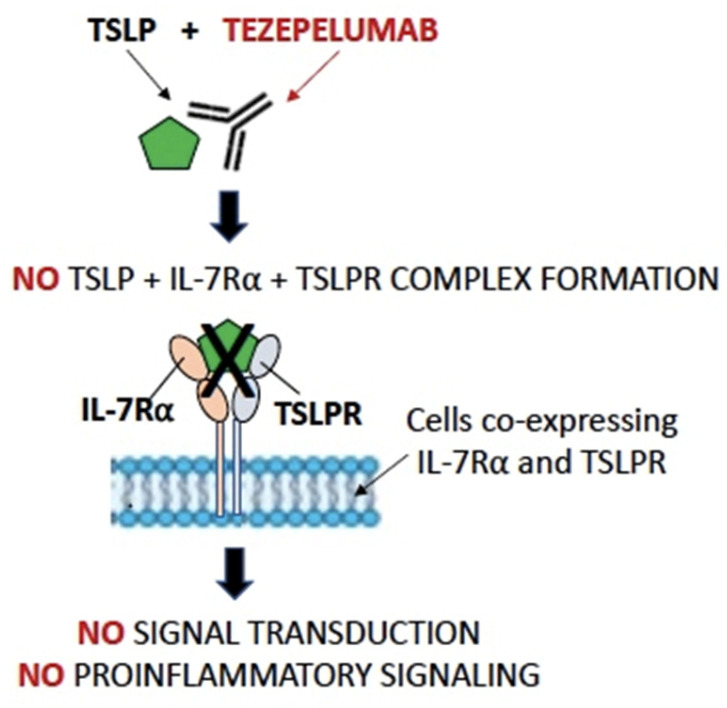
Schematic representation of the Tezepelumab mechanism of action on blocking ternary TSLPR-TSLP-IL-7Rα complex formation and TSLP-related signaling.

As previously said, the first drug developed and marketed against alarmins, particularly TLSP, is Tezepelumab, a human anti-TSLP antibody that prevents TSLP-TSLPR interactions (Figure 2).

### 4.1. Tezepelumab in Asthma

The first disease for which Tezepelumab was tested successfully is asthma. More precisely, Tezepelumab is developed for the severe form of asthma, affecting 5–10% of the whole asthmatic population, usually poorly controllable with medium–high doses of inhaled corticosteroids (ICSs) and a second controller, requiring oral corticosteroids (OCSs) chronically or in short cycles [57]. Despite its role in the control of the disease, the frequent intake of CSs exposes patients to many side effects, both short-term and long-term, including osteoporosis, hyperglycemia, and hypertension [58]. With the aim to reduce OCS side effects, several biologics, including Tezepelumab, have been developed [15]. The effect of Tezepelumab on the reduction in asthma exacerbations was investigated in the phase IIb study “PATHWAY” [59]; the effects on airway inflammation were studied in the phase II study CASCADE [60]; and the OCS-sparing effect was studied in the SOURCE study [61], and efficacy and safety were studied in the NAVIGATOR [62] and DESTINATION [63] studies, all of which are phase III trials. In the PATHWAY study, 550 patients with uncontrolled severe asthma randomized to Tezepelumab or placebo were studied. In the group with Tezepelumab, this could be administered subcutaneously at 70 mg or 210 mg every 4 weeks or at a dosage of 280 mg every 2 weeks, monitoring its efficacy for 52 weeks. At the end of the study, it was shown that Tezepelumab was able to reduce exacerbations at the 70 mg, 4 w dosage by 62% (90% CI 42 to 75; *p* < 0.001); at the 210 mg, 4 w dosage by 71% (90% CI 54 to 82; *p* < 0.001); and at the 280, 2 w dosage by 66% (90% CI 47 to 79; *p* < 0.001), when compared with placebo. Regarding secondary endpoints such as respiratory function, at the 70 mg, 4 w dose, there was a difference of 0.12 L (90% CI 0.02 to 0.22; *p* = 0.015); at the 210 mg, 4 w dose, there was a difference of 0.13 L (90% CI 0.03 to 0.23; *p* = 0.009); and at the 280 mg, 2 w dose, there was a difference of 0.10 L (90% CI 0.05 to 0.25; *p* = 0.002) [59]. In the CASCADE study, the efficacy of the drug was evaluated for moderate–severe asthmatic patients, aged 18 to 75 years, compared with a placebo, and treated for 52 weeks. In this phase 2 study, the investigators aimed to assess the presence of inflammatory cells in the airway submucosa by analyzing bronchial biopsies taken before and after 28 weeks of treatment. The second endpoint of the trial was the change in bronchial hyperactivity, measured with a mannitol test, between the baseline and end-of-treatment values. A total of 116 patients were enrolled in the study and randomized to 210 mg of Tezepelumab or placebo (59 vs. 57) every 4 weeks. A statistically significant (*p* < 0.001) reduction in the eosinophilic infiltrate of the submucosa was reported at the end of treatment, which was greater in the treated patients (ratio of geometric least-square means of 0.15 [95% CI 0.5–0.41]), without a reduction in the other examined cellularity (neutrophils, CD3 T cells, CD4 + T cells, tryptase + mast cells, chymase + mast cells). Regarding the thickness of the basement membrane, no changes were shown between the two treatment groups, either in thickness or in the integrity of the epithelium, at the end of the study. Regarding responsiveness to mannitol, patients in the treatment group demonstrated a significant reduction compared with those who were enrolled in the placebo group [60]. The phase 3 study NAVIGATOR was a double-blind, placebo-controlled trial, in which severe asthmatic patients, aged 12–80 years, were randomized. Patients had to be treated with a medium–high dose of inhaled ICSs associated with a second controller for a period of more than 12 months; they must not have been treated with systemic steroids for at least 3 months before randomization, but they must have had a history of OCS use of more than two times in the previous year. Tezepelumab was administered, as an alternative to placebo, for 52 weeks, at a dosage of 210 mg, subcutaneously, every 4 weeks. The main endpoint of the study was to evaluate the efficacy of the drug in reducing exacerbations, after 52 weeks of treatment, both in patients with eosinophils higher than 300 cells/mcl, and in those with a cellularity below this cut-off. Secondary endpoints were respiratory function, as measured by FEV1; disease control, as measured by ACQ-6; and the impact of the disease on patients’ visual quality, as measured by AQLQ. Of the 1061 randomized patients, 529 were assigned to receive Tezepelumab and 532 were in the placebo group. The rate of exacerbation in the patients treated with Tezepelumab was 0.93 (95% confidence interval [CI], 0.80 to 1.07) and it was 2.10 (95% CI, 1.84 to 2.39) in the group of patients receiving placebo (rate ratio, 0.44; 95% CI, 0.37 to 0.53; *p* < 0.001). In the subsequent analysis of the data from trial, dividing patients according to eosinophils level, in the group of asthmatic patients with less than 150 eosinophils/mcl, the exacerbation rate was 1.02 (95% CI, 0.84 to 1.23) in who received Tezepelumab and 1.73 (95% CI, 1.46 to 2.05) in treated with placebo (rate ratio, 0.59; 95% CI, 0.46 to 0.75; *p* < 0.001). Interesting results were obtained evaluating lung function tests, where an improvement of 0.23 vs. 0.09 L of FEV1 (difference, 0.13 L; 95% CI, 0.08 to 0.18; *p* < 0.001) could be observed in treated patients vs. placebo, after 52 weeks of treatment. The limitations of this analysis included the requirement for patients to have documented historical or on-site reversibility. Additionally, whereas spirometry can capture phenomena occurring in the central airways, benefits in the small airways, improvements in air trapping, or changes in lung volume would require additional methods of measurement, such as plethysmography or oscillometry. Finally, this analysis was not prospectively powered to evaluate significant differences between all the subgroups of patients [62]. SOURCE was a phase 3 double-blind, placebo-controlled study, with the primary endpoint being the evaluation of the efficacy of Tezepelumab in steroid-dependent patients. The randomization was conducted on 150 patients who received 210 mg of Tezepelumab or placebo. At the end of the 48 weeks of observation, the percentage of patients able to reduce OCS intake was similar between the two groups of treatment (odds ratio [OR] 1.28 [95% CI 0.9–2.35], *p* = 0.43); consequently, the primary endpoint was not met. In analyzing patients according to their eosinophils level, using 150 cells/mcl as cut-off, the cumulative odds of efficacy resulted in growths in asthmatic patients exceeding the above-mentioned eosinophils level (2.58 [1.16–5.75] vs. 0.40 [0.14–1.13]) [61]. Adverse events were analyzed in the DESTINATION study, the long-term extension of the above-described NAVIGATOR and SOURCE trials. Observing 528 patients from the NAVIGATOR study, the results show that the incidence of adverse events, over 104 weeks of extension, was 49.62 (95% CI 45.16 to 54.39) per 100 patient-years, compared with the ones receiving placebo with 62.66 (56.93 to 68.81). As for adverse events, only the ones definable as serious were analyzed and an incidence of 7.85 (6.14 to 9.89) per 100 patient-years was established for those who initially received the drug, and 12.45 (9.97 to 15.35) for those who received a placebo. In the SOURCE trial, the incidence of adverse events was 47.15 (36.06 to 60.56) and 69.97 (54.54 to 88.40) per 100 patient-years, respectively, in the Tezepelumab and placebo groups. Concerning serious adverse events, the incidence rates were, respectively, 13.14 (7.65 to 21.04) and 17.99 (10.66 to 28.44) per 100 patient-years, in the Tezepelumab vs. placebo groups [63] (Table 1).

### 4.2. Tezepelumab in CRSwNP

Regarding CRSwNP, eosinophilic CRSwNP (eCRSwNP) and non-eosinophilic (neCRSwNP) are distinct endotypes of CRSwNP and can lead to different clinical outcomes. A meta-analysis suggests that tissue eosinophils may be a useful tool for identifying ECRS, demonstrating that a total eosinophils count (TEC) ≥55/high power field (HPF) can efficiently predict nasal polyp recurrence [64]. A multicenter, randomized, double-blinded, parallel-group, placebo-controlled phase 3 clinical trial evaluating the efficacy and safety of Tezepelumab in severe CRSwNP is now underway: Efficacy and Safety of Tezepelumab in Participants With Severe Chronic Rhinosinusitis With Nasal Polyposis (WAYPOINT) (NCT04851964). Approximately 400 subjects will be randomized globally. Subjects will receive Tezepelumab or a placebo, administered via subcutaneous injection using the accessorized pre-filled syringe (APFS), over a 52-week treatment period. The study also includes a post-treatment follow-up period of 12–24 weeks for participants who complete the 52-week treatment period [65]. Notably, in the DUBHE study, the first phase Ib/IIa study for subjects with uncontrolled CRSwNP, the safety, tolerability, pharmacokinetics, pharmacodynamics, immunogenicity, and preliminary efficacy of multiple ascending doses (MADs) of another anti-TSLP monoclonal antibody, CM326, in patients with eCRSwNP ((TEC) ≥ 55/(HPF)), as well as neCRSwNP (TEC < 55/HPF) will be testified [66].

### 4.3. Tezepelumab in COPD

Currently, the unique clinical trial in COPD is the Tezepelumab COPD Exacerbation Study (COURSE) study, a phase 2a multicenter, randomized, double-blind, placebo-controlled, parallel-group, phase 2a study. The study outcomes are to evaluate the safety and efficacy of Tezepelumab in adults with moderate to very severe chronic obstructive pulmonary disease (COPD) receiving triple-inhaled maintenance therapy and having had two or more documented COPD exacerbations in the 12 months prior to Visit 1 [67].

To date, scientific researchers have noted that type 2 inflammatory disorders often develop in an individual subject in a typical sequential order. This sequence, often referred to as the “atopic march”, highlights the potential role of TSLP and the other epithelial cytokines as initiators and propagators of allergic disease [68]. TSLP is a pleiotropic cytokine, and several cellular targets have been identified, including immune and non-immune cells. The cytokine has been shown to be a key factor in maintaining immune homeostasis and regulating inflammatory responses at mucosal barriers. Various immune cells have been reported to express TSLP receptor (TSLPR), including dendritic cells (DCs), basophils, CD4 + T cells, and group 2 innate lymphoid cells (ILC2s), and disparate cell types have been proposed to have essential role in TSLP-mediated type 2 inflammation [69].

Referring to asthma, Tezepelumab is safe, well tolerated, and provides clinically meaningful improvements in asthma control, including a reduced incidence of exacerbations and hospitalizations in patients with severe asthma. Clinical benefits were associated with reductions in levels of a broad spectrum of cytokines (e.g., interleukin IL-5, IL-13) and baseline biomarkers (e.g., blood eosinophils, immunoglobulin [Ig]E, and fractional exhaled nitric oxide [FeNO]) and were observed across a range of severe asthma phenotypes (i.e., eosinophilic and non-eosinophilic) [16].

Moreover, as previously stated, the data have shown that Tezepelumab is able to produce positive results in terms of reductions in the inflammatory infiltrate in the bronchial site. At the same time, it has been found that the treatment is able to reduce the blood eosinophil count without, however, leading to the depletion of other cell populations of the immune system. This is to be considered an added value among the effects of the drug in efficacy and safety. A new clinical trial is currently underway that will evaluate the effect of anti-TSLP therapy on steroid dependence, due to the fact that, as previously mentioned, the dedicated trial (SOURCE) does not reach the primary endpoint of the OCS-sparing effect of the drug.

With regard to other pathologies of the respiratory tract such as CRSwNP, in recent years, the non-responsive NP to medical and/or surgical treatment was considered a candidate for treatment with monoclonal antibodies. It is evident that a multidisciplinary approach is essential to correctly select patients eligible for treatment, also taking into account comorbidities and monitoring the response to therapy. Preliminary results from ongoing trials on the safety and efficacy of anti-TSLP therapy on these patients seem to be promising.

The administration of biological drugs against TSLP protein has great potential in the treatment of Th2-related diseases such as asthma or CRSwNP in clinical practice and it could be reasonable to state, as for dupilumab, that the therapeutic effect of Tezepelumab administration is currently undergoing study in pathologies not purely th2-mediated, such as COPD. Dupilumab, in fact, is the first and only investigational biologic for chronic obstructive pulmonary disease (COPD) to demonstrate a significant 30% reduction in acute, moderate, or severe exacerbations compared to placebo, based on data from the BOREAS phase 3 study [70].

Despite all currently approved biological treatments being delivered intravenously or subcutaneously, promising results come from clinical studies conducted on the administration of ecleralimab, a potent neutralizing antibody fragment (fragment antigen-binding (F_ab_)) directed against human TSLP that has been developed for inhalation. These results suggest that ecleralimab may be a promising, new therapeutic class for asthma treatment [71]. Due to ongoing clinical trial results, actual knowledge of TSLP’s physiological role in health and diseases and the use of economic and human resources in the study of TSLP as a target and in the development of the related targeted biological therapies appears to be a useful investment in order to increase the resources available in clinical practice in order to guarantee patients an increasingly personalized and effective therapy.

## 5. Conclusions

In conclusion, the possibility of acting on a different target, than those available to date, certainly provides an advantage from a therapeutic point of view. Acting on the alarmin cascade and in particular on what occurs at the epithelial level could prospectively lead to undoubted advantages, such as the regeneration of the epithelial barrier. Real-life data and post-marketing observations of anti-TSLPs may give further insights regarding the effectiveness of this target in the respiratory disease setting.

## 6. Future Directions

The increased understanding of the inflammatory mechanisms associated with type 2 pathologies has led to the development of a series of drugs capable of targeting some of these pathophysiological mechanisms. However, until now, the management of these pathologies has been compartmentalized, with specialists focusing on the “dominant pathology”. Unfortunately, some forms related to the inflammatory pathway have often been misrecognized. With the advent of drugs capable of blocking various points in the inflammatory mechanisms, the future direction should involve a multidisciplinary approach to patients. This approach aims to not only investigate the specific pathology for which the patient sought the specialist but also explore other comorbidities related to inflammation. The goal is to uncover any potential underlying pathology and establish the most personalized therapy possible. Crucial to this shift is the establishment of multidisciplinary teams to discuss patients, compare notes, and formulate common pathways for all individuals affected by potential type 2 diseases. Advancements in understanding the products of inflammation related to epithelial stress and their potential impact on various pathologies undoubtedly enhance and broaden the potential interventions for different diseases.

## Figures and Tables

**Table 1:** Main outcomes of principal clinical trials in severe asthma.

	Design	Exacerbations Annualized Rate of Exacerbations/Relative Reduction vs. Placebo	OCS	FEV1 (L) (Difference vs. Placebo)	Control of Disease (Difference vs. Placebo, Evaluated with ACQ6)	Outcomes
ASTHMA						
PATHWAY	70 mg sc Q4w 210 mg sc Q4w 280 mg sc Q2w Placebo	0.27 (0.20 to 0.36)/62 (42 to 75) 0.20 (0.14 to 0.28)/71 (54 to 82) 0.23 (0.17 to 0.32)/66 (47 to 79) 0.72 (0.61 to 0.86)	Not assessed	+0.12 (0.02 to 0.22) +0.13 (0.03 to 0.23) +0.15 (0.05 to 0.25)	−0.26 (−0.52 to 0.01) −0.29 (−0.56 to −0.01) −0.31 (−0.58 to −0.04)	Exacerbation reduction. Respiratory function improvement
NAVIGATOR	210 mg sc Q4W Placebo	0.93 (95% CI 0.80 to 1.07) 2.10 (95% CI 1.84 to 2.39)	Not assessed	+0.13 (0.08 to 0.18)	−0.33 (−0.46 to −0.20)	Exacerbation reduction. Respiratory function improvement.
SOURCE	210 mg Q4W Placebo	1.38 (0.98 to 1.95) 2.00 (1.46 to 2.74) Annualized rate of exacerbations	1.28 (0.69–2.35) Cumulative OR	+0.26 (0.13 to 0.39)	−0.37 (0.71 to −0.02)	No differences in OCS intake.

## Data Availability

The data are contained within the article.
